# Interaction of a four-level atom with a quantized field in the presence of a nonlinear Kerr medium

**DOI:** 10.1038/s41598-024-51649-9

**Published:** 2024-01-11

**Authors:** S. Almalki, K. Berrada, S. Abdel-Khalek, H. Eleuch

**Affiliations:** 1 Department of Physics, Najran University, Najran, Saudi Arabia; 2 Department of Physics, College of Science, Imam Mohammad Ibn Saud Islamic University (IMSIU), Riyadh, Saudi Arabia; 3 The Abdus Salam International Centre for Theoretical Physics, Strada Costiera 11, 34151 Trieste, Italy; 4 Department of Mathematics and Statistics, College of Science, Taif University, P.O. Box 11099, 21944 Taif, Saudi Arabia; 5 Department of Applied Physics and Astronomy, University of Sharjah, 27272 Sharjah, United Arab Emirates; 6 College of Arts and Sciences, Abu Dhabi University, 59911 Abu Dhabi, United Arab Emirates; 7 Institute for Quantum Science and Engineering, Texas A&M University, College Station, TX 77843 USA

**Keywords:** Physics, Information theory and computation, Optical physics, Quantum physics

## Abstract

Quantum entanglement and atomic coherence are examined for a system consisting of a four-level atom (FLA) interacted with a nonlinear quantum field. We assume that the FLA-field coupling, Kerr medium and quantified field are all $f$-deformed with full nonlinear formalism. We consider N-configuration and cascade (C)-configuration of the FLA. We explore the impact of field deformation and Kerr medium on the dynamics of the quantumness measures when the quantized field is initially prepared in a deformed coherent state without and with Kerr medium effect. Moreover, we examine the statistical properties of the radiation field using the second order correlation function. The results indicate how the considered quantumness measures in the FLA–field system can be manipulated and controlled through the parameters of the quantum model.

## Introduction

Quantum electrodynamics (QED) has been considered as crucial area of study in quantum optics, due to the inherently interesting and far-reaching implications resulting from the exploration of the fundamental characteristics of light-matter interaction^1^. In a cavity QED, the typical representation is that an atom interacts with a single near-resonant quantized mode of the electromagnetic field. Therefore, the model describes coupling between a two-level particle and a harmonic oscillator. The study of the interaction is analytically obtained by the solvable Jaynes-Cummings model (JCM)^2,3^. This simple model theoretically explains many nonclassical phenomena, including collapse-revivals^4^, Rabi oscillations^5^, and entanglement^6^. It also performs an essential role in quantum information processing^7–9^. Additionally, the quantum described by JCM is one of several possible methods for the generation of nonclassical states^10,11^. The reduced number of degrees of freedom in this model facilitates the confirming of its dynamics by experiments with the Rydberg atom in high-quality cavities^12^.

The extensions of the JCM are becoming increasingly intriguing especially with the advancement of extant experimental techniques. There are some theoretical proposals considering more complex systems; multiple atoms^13,14^, multilevel atoms^15,16^, multi-mode field^17^, or considering nonlinear optical process such as Kerr nonlinearity^18^. In addition, the dynamics of two-level atom have been considered with intensity-dependent coupling^19^. It implies that the intensity-dependent coupling appears to be a more practical solution to the problem of atom–field interaction, particularly in the domain of strong coupling where the rotating wave approximation (RWA) fails^20,21^.

In other respects, there are unavoidable two dissipative mechanisms affect atoms in real cavities: spontaneous emission and the loss of energy from the cavity. These mechanisms are not introduced in the original JCM; however, experimental test of the JCM and its generalizations have been enhanced by the including damping mechanisms and provides precise verifications^22^. Regarding the nonlinear properties of light-matter interaction, the Kerr effect has been utilized in several noteworthy applications, including quantum non-demolition measurements^23^, quantum fluctuations^24^, the generation of entangled macroscopic quantum states^25^, and quantum information processing^26^. Despite the fact the natural Kerr effect is small, it can be enhanced via atomic coherence^27^, quantum interference^28^, and electromagnetically induced transparency^29^. In addition, the atom–field coupling can be considered in terms of the light intensity. In this case, the coupling is known as intensity-dependent coupling, and the quantized field is described by nonlinear coherent states (CSs)^30^.

There are several atomic configurations for FLAs and they can be realized experimentally through rubidium atoms^31–37^. FLAs have been considered to examine several physical effects such as Doppler-free spectroscopy^38^, spontaneous and stimulated Ramann processes^39^, coherent trapping^40,41^ and two-photon lasers^42^, etc. The four-state atom in N-configuration is the simplest model used to explain the EIA resonance^43^. The experimental observations of the large Kerr nonlinearity in the four-level electromagnetically induced transparency scheme have been introduced, where the experiments were performed by cold ^87^Rb atoms confined in a magneto-optical trap^44^. The cross-Kerr Hamiltonian for the FLA coupled to electromagnetic fields in the context of N-configuration, by using the hyperfine components of the ^87^Rb in order to form the FLA, has been studied^45^. An ensemble of ^87^Rb atoms in the context of FLAs trapped within a magneto-optical trap with similar conditions to recent experiments has been considered^44^. The experimental observations have showed that the laser-induced population transfers among the levels of N four-level schemes, considering alkali-metal atoms, are incoherently produced by spontaneous emission processes and that the explored incoherent N schemes are transformed into coherent ones through applying a resonant laser radiation^46^. More recently, the physical origin of the interferences was explored considering four-level cascade rubidium atoms through simultaneous interactions with three energies; one radio frequency field and two optical fields^47^.

Based on the above considerations, the aim of this work is to examine the quantum entanglement, coherence and statistical properties in a quantum system consists of an FLA coupled with a nonlinear field in the presence of a Kerr medium. We assume that the FLA-field coupling, Kerr medium and quantified field are all $f$-deformed with full nonlinear formalism. We consider the N- and C-configuration of the FLA. We illustrate the impact of field deformation and Kerr medium on the dynamics of the quantumess measures when the radiation field is initially considered in a deformed coherent without and with Kerr medium effect. Moreover, we explain how the quantum resources can be manipulated and controlled through the parameters of the quantum model. The following is structured as follows. In section "Quantum model and dynamics", we present the model which describes the FLA-one mode field system and its quantum dynamics. The wave function of the atomic system under consideration may now be determined. Section "Quantum quantifiers and numerical results", discusses the dynamical properties of the proposed quantum phenomena under the considering the impact of the model parameters. After that, a summary of the findings and some conclusions will be presented.

## Quantum model and dynamics

**Figure 1:**
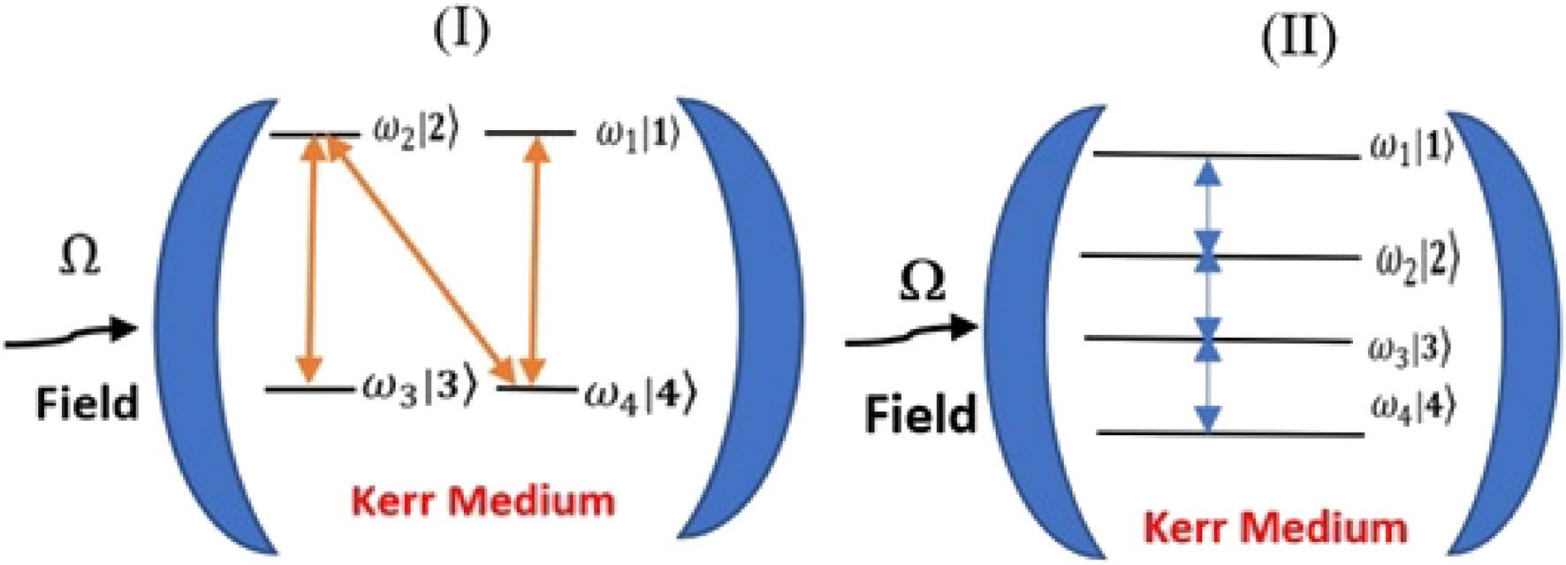
Transition scheme of a FLA $|k\rangle$; $k$= 1, 2, 3, 4 interacting with a one mode field with frequency $\Omega$ in the presence of Kerr medium. Label (I) is for the N-configuration and label (II) corresponds to the C-configuration.

We introduce two quantum schemes of a FLA, considering N- and C-configuration, as illustrated in Fig. 1, interacting with a quantized field oscillating with a frequency $\Omega$ and initially described in a deformed coherent state (DCS). We consider that the FLA with energies ${\omega }_{k} (k=\mathrm{1,2},\mathrm{3,4})$ corresponding to an atomic state from the upper state $\left|1\right.\rangle$ to the lower state $\left|4\right.\rangle$. The states $|k\rangle$ are ordered in N-configuration with the transitions $\left|1\right.\rangle \to \left|4\right.\rangle , \left|2\right.\rangle \to \left|4\right.\rangle$ and $|2\rangle \to |3\rangle$, while for C-configuration $|k\rangle$ are ordered as $\left|1\right.\rangle \to \left|2\right.\rangle ,$ $|2\rangle \to |3\rangle$ and $\left|3\right.\rangle \to \left|4\right.\rangle .$ Based on the RWA, the FLA Hamiltonian for N- and C-configuration are denoted by ${\widehat{H}}_{{\text{NC}}}$ and ${\widehat{H}}_{{\text{CC}}}$, respectively, and can be written in the presence of Kerr medium effect as^48^

$$ \hat{H}_{NC} = \Omega \hat{R}^{\dag } \hat{R} + \mathop \sum \limits_{k = 1}^{4} \omega_{k} \left| {k\rangle \langle k} \right| + \chi \hat{R}^{\dag 2} \hat{R}^{2} + \lambda \left\{ {\hat{R}\left( {\lambda_{1} \left| {1\rangle \langle 4} \right| + \lambda_{2} \left| {2\rangle \langle 3} \right| + \lambda_{3} \left| {2\rangle \langle 4} \right|} \right) + h.c} \right\}, $$

tends to

1 $$ \hat{H}_{CC} = \Omega \hat{R}^{\dag } \hat{R} + \mathop \sum \limits_{k = 1}^{4} \omega_{k} \left| {k\rangle \langle k} \right| + \chi \hat{R}^{\dag 2} \hat{R}^{2} + \lambda \left\{ {\hat{R}\left( {\lambda_{1} \left| 1 \right.2\left| { + \lambda_{2} } \right|23\left| { + \lambda_{3} } \right|3\left. 4 \right|} \right) + h.c} \right\}. $$

Here $\lambda$ is the parameter describing the coupling between the FLA and the nonlinear field, and χ Kerr medium parameter. The operators $\hat{R}^{\dag }$ and $\widehat{R}$ represent the $f$-deformed creation and annihilation operators, respectively, constructed from the usual bosonic operators $\hat{a}^{\dag }$ and $\hat{a}$ as $\hat{R} = \hat{a}f\left( {\hat{n}} \right)$ and $\hat{R}^{\dag } = f\left( {\hat{n}} \right)\hat{a}^{\dag }$ with $\hat{n} = \hat{a}^{\dag } \hat{a}$ is the number operator and $f$ is a characteristic function of the deformation determining the field nonlinearity. The operators $\hat{R}^{\dag }$ and $\hat{R}$ verify the bosonic commutation relations

2 $$ \begin{gathered} \hat{R}\hat{R}^{\dag } - \hat{R}^{\dag } \hat{R} = \left( {\hat{n} + 1} \right)f^{2} \left( {\hat{n} + 1} \right) - \left( {\hat{n}} \right)f^{2} \left( {\hat{n}} \right) \hfill \\ \left[ {\hat{R},\hat{n}} \right] = \widehat{R, }{ }\left[ {\hat{R}^{\dag } ,\hat{n}} \right] = - \hat{R}^{\dag } , \hfill \\ \end{gathered} $$

and they act on the Fock states as

3 $$ \hat{R}^{\dag } \left| n \right. = f\left( {{\text{n}} + 1} \right)\sqrt {n + 1} \left| {n + 1} \right.,\hat{D}\left| k \right. = f\left( {\text{n}} \right)\sqrt {\text{n}} \left| {k - 1} \right.. $$

It should be emphasized that selecting various nonlinearity functions results in various Hamiltonian systems, which may then lead to various physical outcomes. In the case of $f\left(\widehat{n}\right)=1$, the Hamiltonian defined in Eq. (1) represents the usual generalized JCM with Kerr nonlinearity and the quantum algebra (2) becomes the Heisenberg-Weyl algebra described by $\hat{a}^{\dag }$, $\hat{a}$ and the identity operator ${\text{I}}$.

In analogy to the Glauber states, the ${\text{DCSs}}$ are therefore defined as the eigenvectors of $\widehat{{\text{R}}}$:

4 $$\widehat{R}|\alpha ,p\rangle =\alpha |\alpha ,p\rangle ,$$

where $\alpha$ is a complex eigenvalue and $p$ is a real that represents the deformed parameter. The $DCSs$ are given by

5 $$|\alpha ,p\rangle =\frac{1}{\sqrt{{{\text{exp}}}_{p}\left[|\alpha {|}^{2}\right]}}\sum_{n=0}^{\infty }\frac{{\alpha }^{n}}{\sqrt{[n{]}_{p}!}}|n\rangle ,$$

and we have introduced

6 $${{\text{exp}}}_{p}\left[x\right]=\sum_{k=0}^{\infty }\frac{{x}^{k}}{{\left[k\right]}_{p}!}, {\left[k\right]}_{p}!=\left[k{f}^{2}(k)\right]\times \left[(k-1){f}^{2}(k-1)\right]\times \cdot \cdot \cdot \times \left[{f}^{2}(1)\right].$$

The function $ex{p}_{p}$ is a deformed version of the ordinary exponential function. They become coincident when $f$ tends to unity. The characteristic function of the deformation is defined by^49–51^

7 $${f}^{2}\left(k\right)=\frac{1}{k}\frac{{p}^{1+{\text{k}}}-{p}^{1-{\text{k}}}}{{p}^{2}-1}.$$

Note that ${{\text{exp}}}_{p}[x]{{\text{exp}}}_{p}[y]\ne {{\text{exp}}}_{p}[x+y]$; i.e., we have a non-extensive exponential that is found in many physical problems. Clearly $f(k)=1$ when $p\to 1$ and DCSs become the standard CSs.

We assume that the FLA begins from its ground state $|4\rangle$ and the quantized field from the DCS. The wave function corresponding to the quantum Hamiltonians (1) and (2) at any time $T>0$ can be formulated as

8 $${\left|{\text{U}}\left(T\right)\right.\rangle }_{NC}=\sum_{k=0}^{\propto }{Q}_{k}[\left({A}_{1}\left(k,T\right)\left|1,k\right.\rangle +{A}_{2}\left(k,T\right)\left|2,k\right.\rangle \right)+ {A}_{3}(k,T)|3,k+1\rangle +{A}_{4}(k,T)|4,k+1\rangle ],$$

9 $${\left|{\text{U}}\left(T\right)\right.\rangle }_{CC}=\sum_{k=0}^{\approx }{Q}_{k}[\left({A}_{1}\left(k,T\right)\left|1,k\right.\rangle +{A}_{2}\left(k,T\right)\left|2,k+1\right.\rangle \right)+ {A}_{3}(k,T)|3,k+2\rangle +{A}_{4}(k,T)|4,k+3\rangle ],$$

where $T=\lambda t\mathrm{ is the scaled time}$ and the coefficients ${A}_{j}$ are the probability amplitudes, which can be obtained by solving the time-dependent Schrödinger equation $i\frac{d}{dT}{|{\text{U}}({\text{T}})\rangle }_{{\text{NC}}}\left({|{\text{U}}({\text{T}})\rangle }_{{\text{CC}}}\right)={\widehat{H}}_{{\text{NC}}}\left({\widehat{H}}_{{\text{CC}}}\right)|U(T)\rangle$ with the initial state $\left|{\text{U}}\left(0\right)\right.\rangle =|\alpha ,p,4\rangle$. Therefore, the coefficients ${A}_{j}$ obey the following coupled system of ODEs:

10 $$\frac{d}{dT}\left(\begin{array}{l}{A}_{1}\\ {A}_{2}\\ {A}_{3}\\ {A}_{4}\end{array}\right)={\mathrm{\rm M}}_{{\text{CC}}}\left({\mathrm{\rm M}}_{{\text{NC}}}\right)\left(\begin{array}{l}{A}_{1}\\ {A}_{2}\\ {A}_{3}\\ {A}_{4}\end{array}\right),$$

where

11 $${\mathrm{\rm M}}_{{\text{CC}}}=\left(\begin{array}{llll}-\mathrm{i\chi }k(k-1)& -i\lambda \sqrt{k+1}& 0& 0\\ -i\lambda \sqrt{k+1}& -\mathrm{i\chi }k({\text{k}}+1)& -i\lambda \sqrt{k+2}& 0\\ 0& -i\lambda \sqrt{k+2}& -\mathrm{i\chi }(k+1)({\text{k}}+2)& -i\lambda \sqrt{k+3}\\ 0& 0& -i\lambda \sqrt{k+3}& -\mathrm{i\chi }(k+2)({\text{k}}+3)\end{array}\right),$$

and

12 $${\mathrm{\rm M}}_{{\text{NC}}}=\left(\begin{array}{llll}-\mathrm{i\chi }k(k+1)& 0& 0& -i\lambda \sqrt{k+1}\\ 0& -\mathrm{i\chi }k(k+1)& -i\lambda \sqrt{k+1}& -i\lambda \sqrt{k+1}\\ 0& -i\lambda \sqrt{k+1}& -\mathrm{i\chi }k({\text{k}}-1)& 0\\ -i\lambda \sqrt{k+1}& -i\lambda \sqrt{k+1}& 0& -\mathrm{i\chi }k(k-1)\end{array}\right).$$

Based on the FLA-field wave function $|{\text{U}}(T)\rangle$, we can extract the time-dependent properties of various quantum phenomena that are associated with the proposed system. Here, we consider the population inversion, quantum coherence and von Neumann entropy, which depend on the the density matrix elements of ${\rho }^{{\text{FLA}}}(t)$:

13 $${\rho }^{{\text{FLA}}}(T)=T{r}_{field}|{\text{U}}(T)\rangle \langle {\text{U}}(T)|=\sum_{j=1}^{4}\sum_{l=1}^{4}{\rho }_{jl}(T)|j\rangle \langle l|.$$

## Quantum quantifiers and numerical results

In order to display the influence of Kerr medium and deformation of the field on the quantum quantifiers, in Figs. 1, 2, 3, 4, 5, 6, 7 and 8, we show the temporal evolution of the atomic inversion, second-order correlation function, atomic entropy and atomic coherence in both cases of N- and C-configuration.

**Figure 2:**
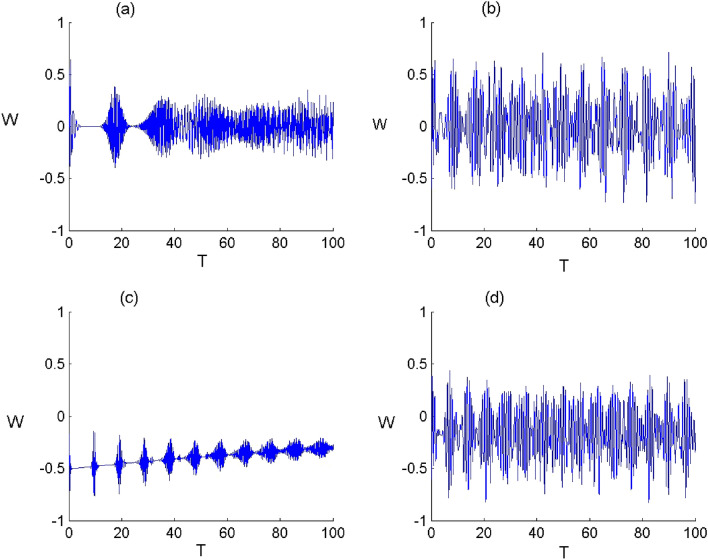
Dynamics of the atomic inversion $W={\rho }_{11}-{\rho }_{44}$ of FLA-NC interacting with the field initially in the DCS with $\alpha =\sqrt{20}$ and for the parameter values of (Deformation, Kerr) designed by $\left(p,\chi \right)$ as: (**a**) $(p,\chi )=(\mathrm{1,0})$, (**b**) $(p,\chi )=(\mathrm{2.5,0})$, (**c**) $(p,\chi )=(\mathrm{1,0.3})$ and (**d**) $(p,\chi )=(\mathrm{2.5,0.3})$.

**Figure 3:**
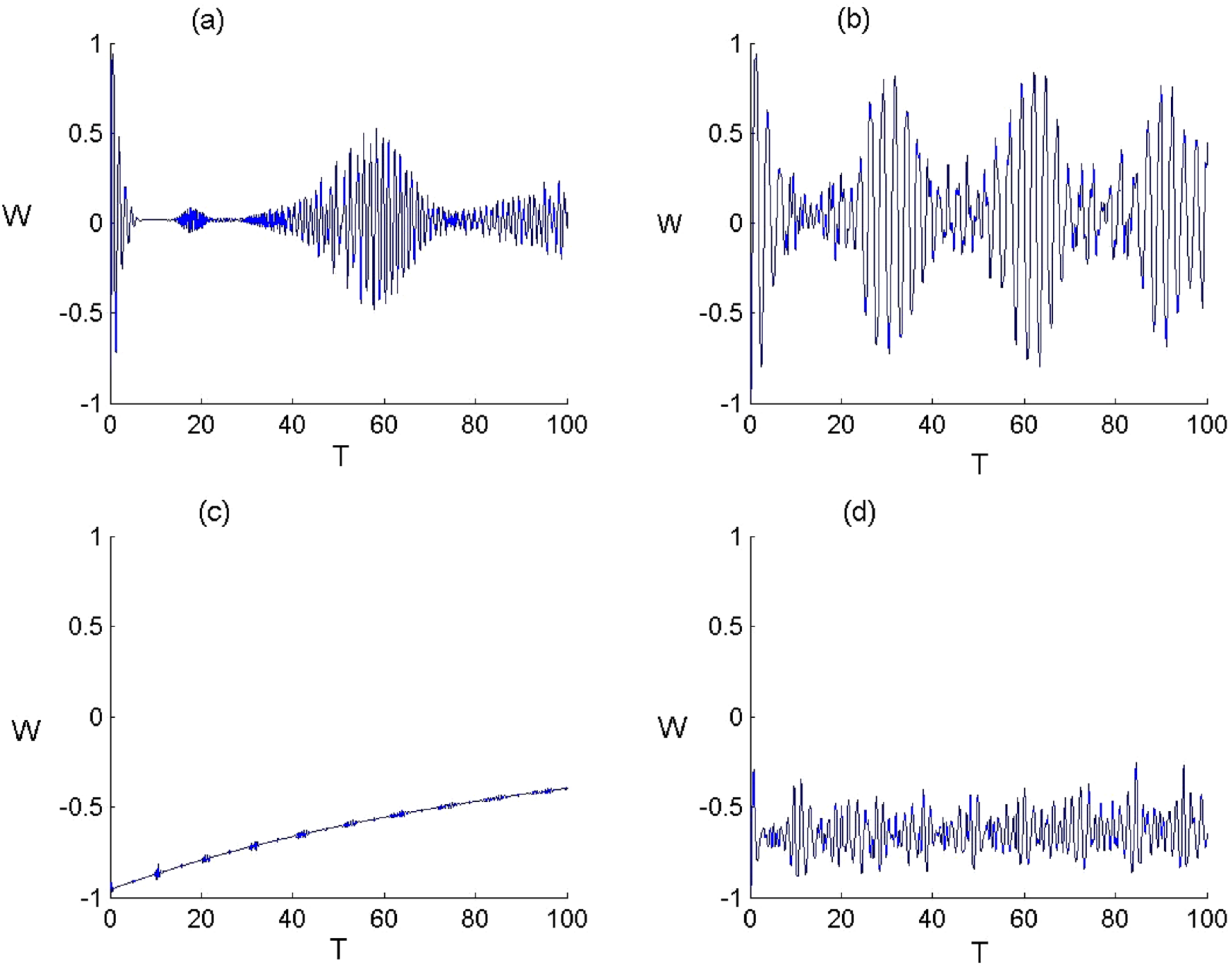
Dynamics of the atomic inversion $W={\rho }_{11}-{\rho }_{44}$ of FLA-CC interacting with the field initially in the DCS with $\alpha =\sqrt{20}$ and for the parameter values of (Deformation, Kerr) designed by $\left(p,\chi \right)$ as: (**a**) $(p,\chi )=(\mathrm{1,0})$, (**b**) $(p,\chi )=(\mathrm{2.5,0})$ , (**c**) $(p,\chi )=(\mathrm{1,0.3})$ and (**d**) $(p,\chi )=(\mathrm{2.5,0.3})$.

**Figure 4:**
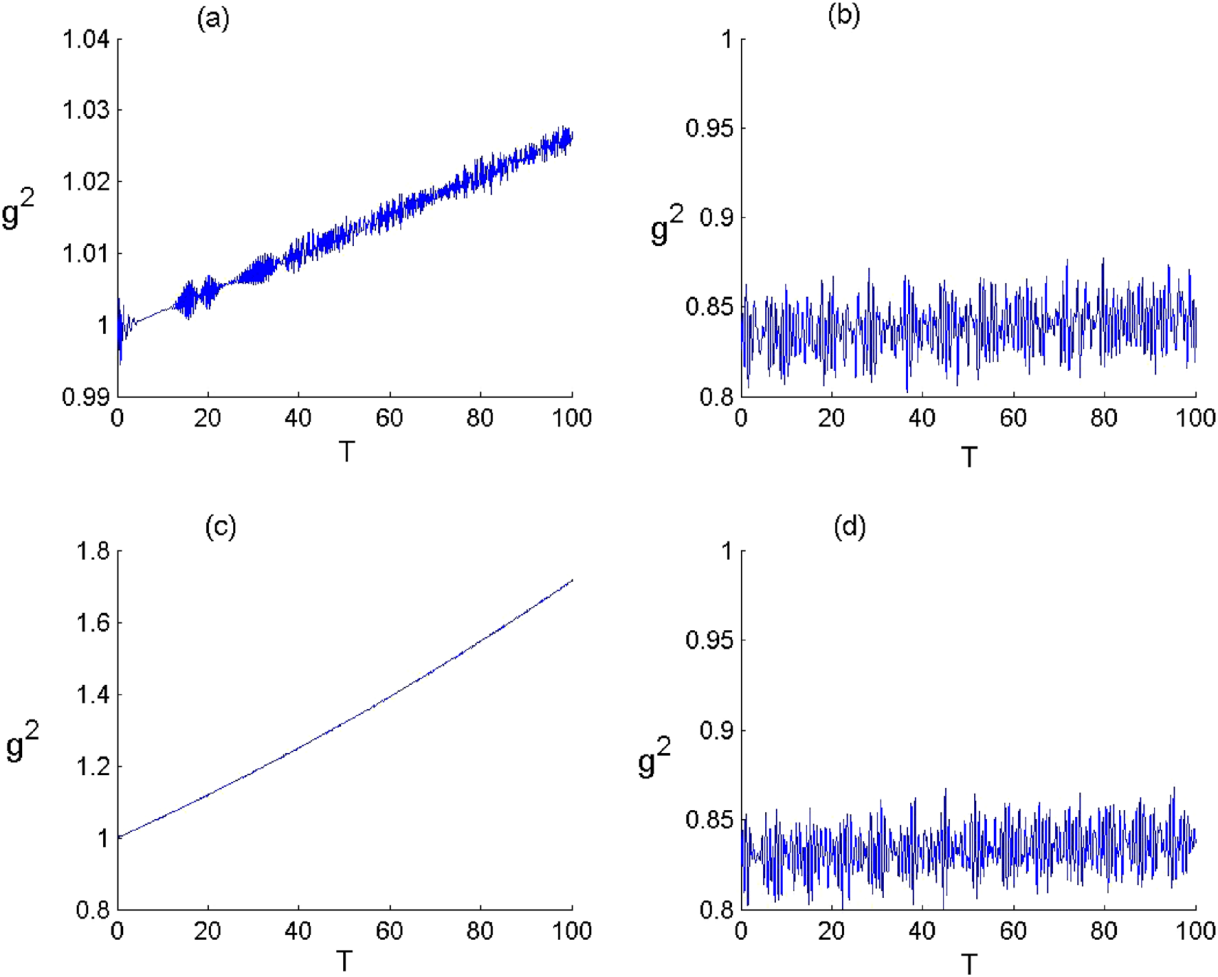
Dynamics of the second order correlation ${g}^{2}$ of FLA-NC interacting with the field initially in the DCS with $\alpha =\sqrt{20}$ and for the parameter values of (Deformation, Kerr) designed by $\left(p,\chi \right)$ as: (**a**) $(p,\chi )=(\mathrm{1,0})$, (**b**) $(p,\chi )=(\mathrm{2.5,0})$, (**c**) $(p,\chi )=(\mathrm{1,0.3})$ and (**d**) $(p,\chi )=(\mathrm{2.5,0.3})$.

**Figure 5:**
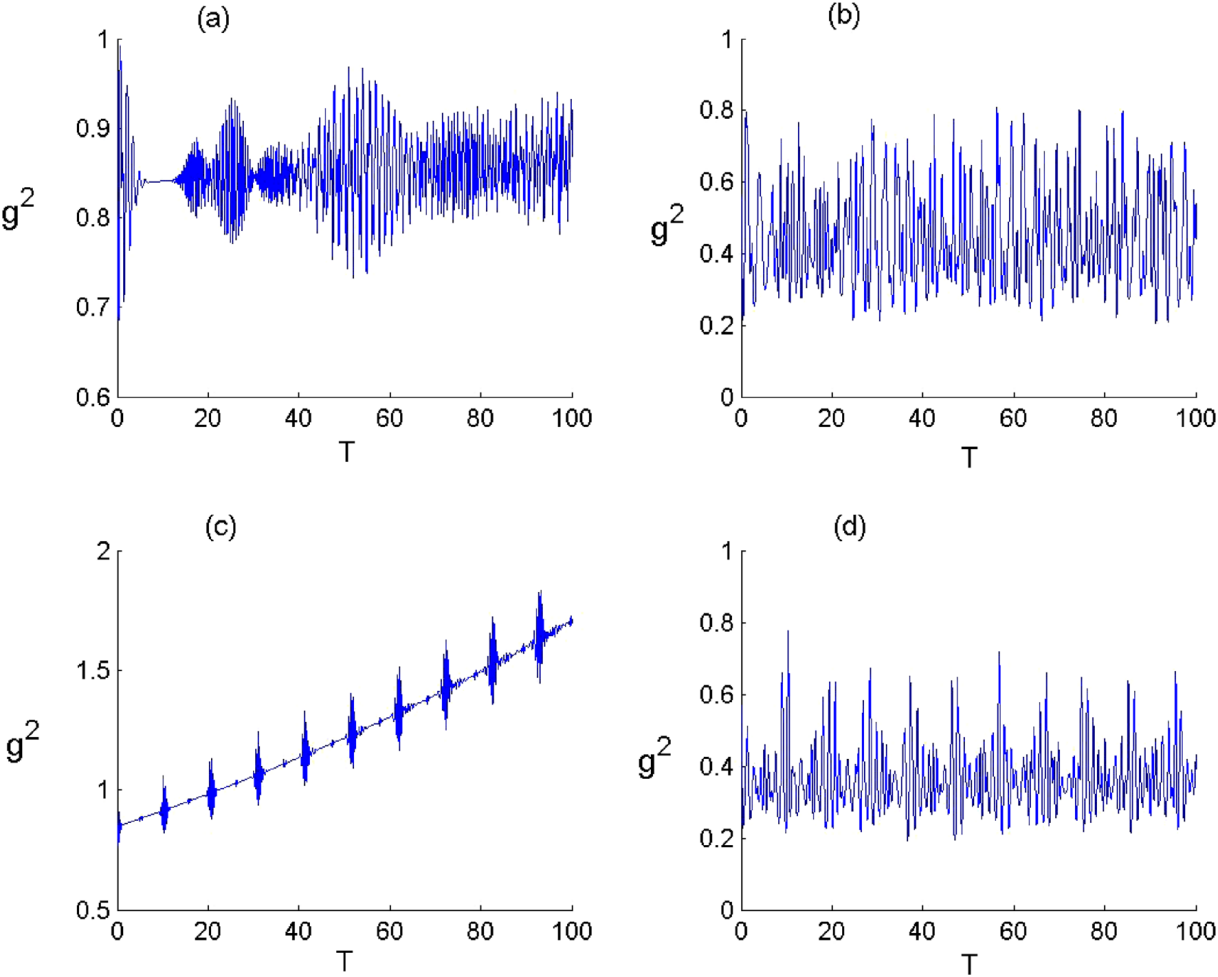
Dynamics of the second order correlation ${g}^{2}$ of FLA-CC interacting with the field initially in the DCS with $\alpha =\sqrt{20}$ and for the parameter values of (Deformation, Kerr) designed by $\left(p,\chi \right)$ as: (**a**) $(p,\chi )=(\mathrm{1,0})$ , (**b**) $(p,\chi )=(\mathrm{2.5,0})$, (**c**) $(p,\chi )=(\mathrm{1,0.3})$ and (**d**) $(p,\chi )=(\mathrm{2.5,0.3})$.

**Figure 6:**
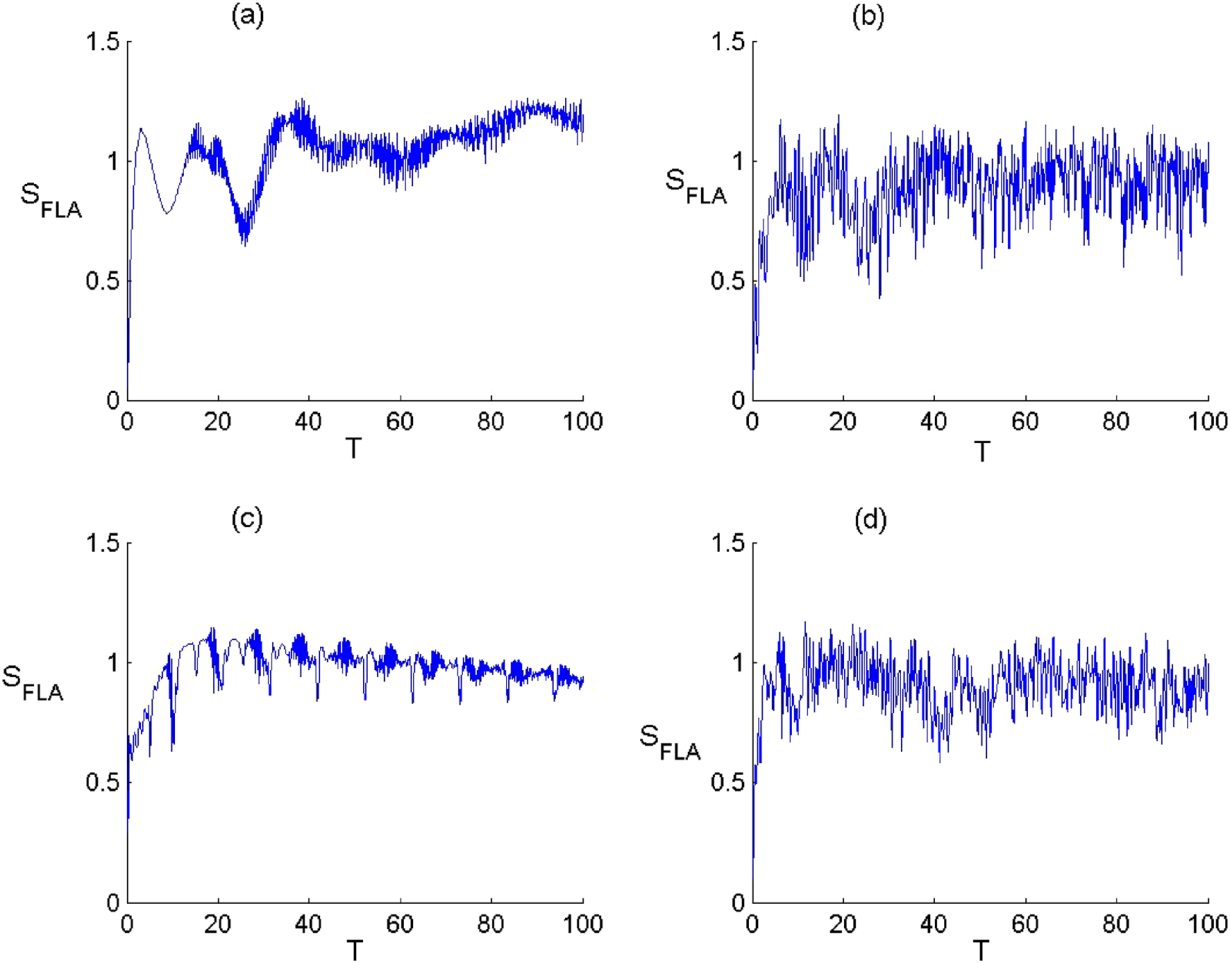
Dynamics of the FLA-NC Neumann entropy when the field initially in the DCS with $\alpha =\sqrt{20}$ and for the parameter values of (Deformation, Kerr) designed by $\left(p,\chi \right) as$: (**a**) $(p,\chi )=(\mathrm{1,0})$ , (**b**) $(p,\chi )=(\mathrm{2.5,0})$, (**c**) $(p,\chi )=(\mathrm{1,0.3})$ and (**d**) $(p,\chi )=(\mathrm{2.5,0.3})$.

**Figure 7:**
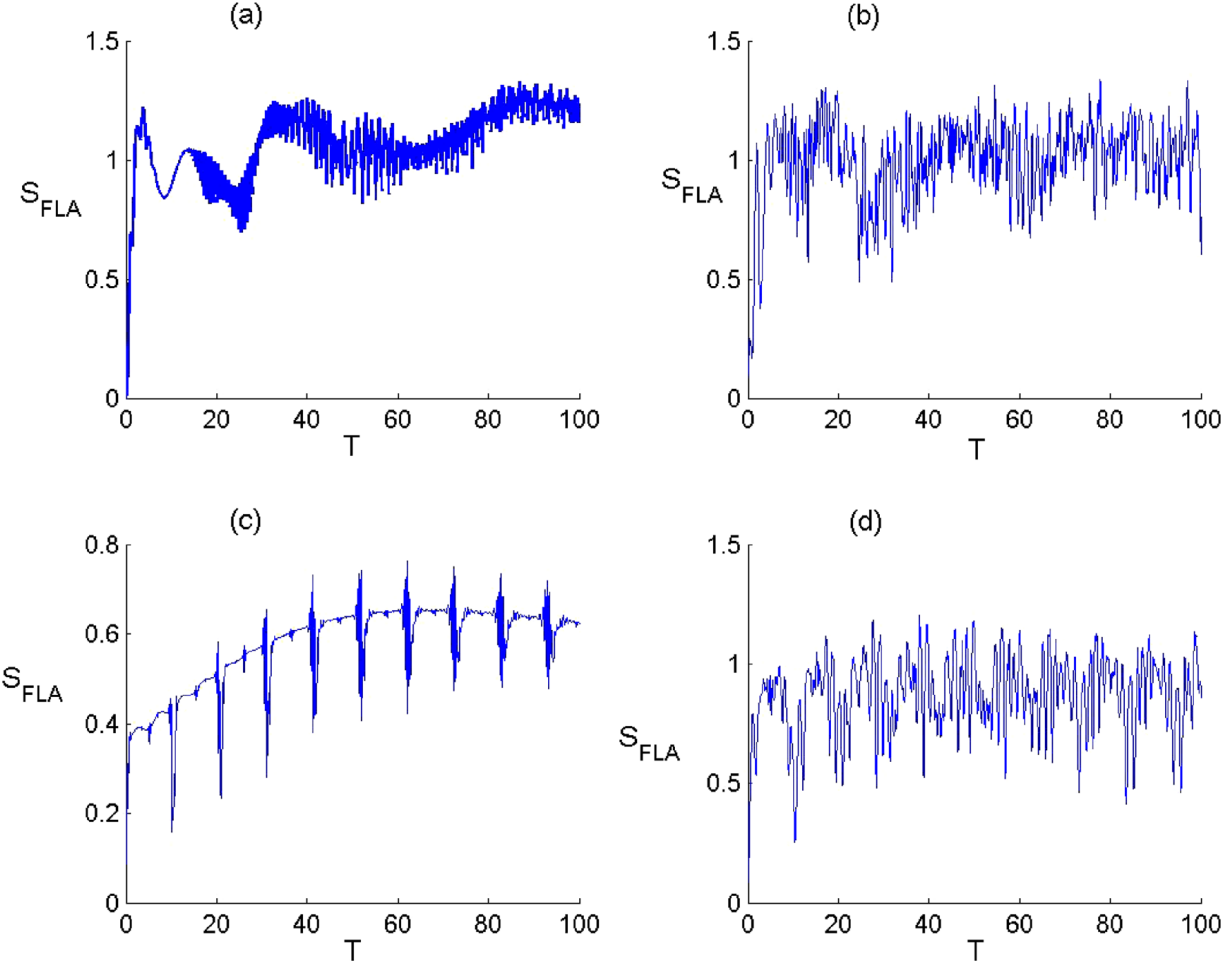
Dynamics of the FLA-CC von Neumann entropy when the field initially in the DCS with $\alpha =\sqrt{20}$ and for the parameter values of (Deformation, Kerr) designed by $\left(q,\chi \right) as$: (**a**) $(p,\chi )=(\mathrm{1,0})$ , (**b**) $(p,\chi )=(\mathrm{2.5,0})$, (**c**) $(p,\chi )=(\mathrm{1,0.3})$ and (**d**) $(p,\chi )=(\mathrm{2.5,0.3})$.

**Figure 8:**
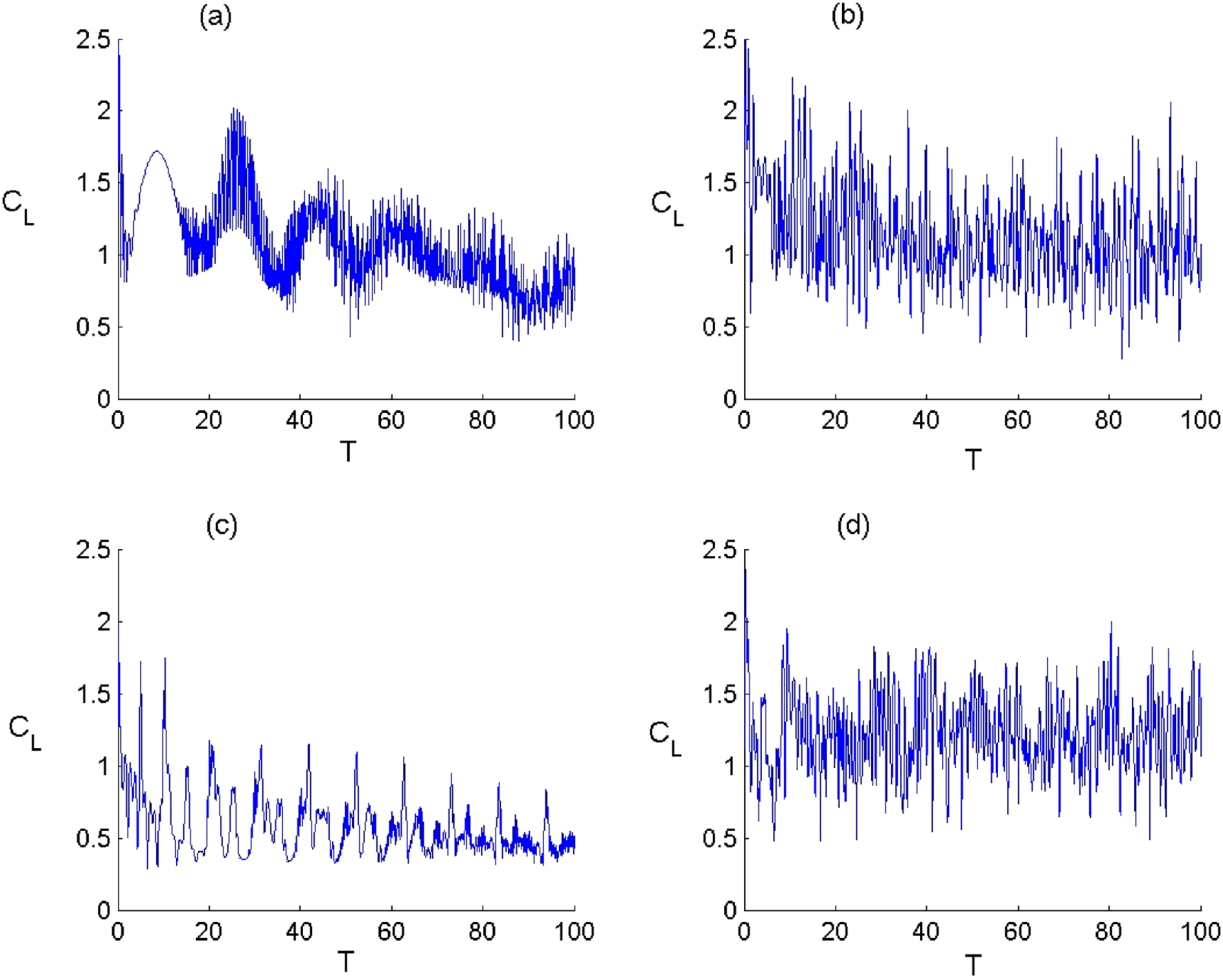
Dynamics of the quantum coherence ${C}_{L}$ of FLA-NC interacting with the field initially in the DCS with $\alpha =\sqrt{20}$ and for the parameter values of (Deformation, Kerr) designed by $\left(p,\chi \right) as$: (**a**) $(p,\chi )=(\mathrm{1,0})$ , (**b**) $(p,\chi )=(\mathrm{2.5,0})$, (**c**) $(p,\chi )=(\mathrm{1,0.3})$ and (**d**) $(p,\chi )=(\mathrm{2.5,0.3})$.

### Population inversion

In the view of quantum optics and information, one of the most important quantities is the population inversion^52–55^. This quantity can be utilized to identify the times of collapse and revival that are significant in determining the periods of separable and maximally states. The atomic population inversion of ${\rho }^{{\text{FLA}}}\left(t\right)$ is defined by

14 $$W={\rho }_{11}\left(T\right)-{\rho }_{44}\left(T\right).$$

In Figs. 2 and 3, we show the time variation of the atomic inversion of the two configurations for various values of $p$ and $\chi$. Generally, the dynamical behavior of the atomic inversion function is largely affected by the parameters $q$ and $\chi$ as well as the atomic configuration. In the limit of $p\to 1$ and $\upchi \to 0$, we obtain that the atomic inversion exhibits oscillations with revival and collapse phenomena. The presence of field deformation ($p\to 2.5$ and $\upchi \to 0$) leads to organize the behavior of $W$ and enhance its oscillations amplitude during the dynamics. Whereas the existence of the Kerr medium ($p\to 1$ and $\upchi \to 0.3$) leads to decrease the amplitude of oscillations of the function $W.$ Furthermore, we can mention that the oscillations of the function $W$ and its amplitudes depend on the N- and C-configuration. When the field deformation and Kerr medium are considered ($p\to 2.5$ and $\upchi \to 0.3$), The function $W$ randomly oscillates between the excited state and the ground state during the considered interaction period. From these results, we conclude that the deformation of field can enhance the amplitude of oscillations in the atomic inversion and that the presence of the Kerr medium leads to organize the behavior of the measure of the atomic inversion accompanied with a diminution in the oscillations amplitudes.

### Nonclassical effects

The second-order correlation function is widely utilized to examine the effects of bunching or antibunching^56^, which is defined as follows

15 $${g}^{(2)}(\tau )=\frac{\langle :I\left(t\right)I\left(t+\tau \right):\rangle }{\langle I(t){\rangle }^{2}},$$

where $I$ represents the field intensity with $:I(t)I(t+\tau ):={R}_{+}^{q}(t){R}_{+}^{q}(t+\tau ){R}^{q}(t+\tau ){R}^{q}(t).$ The function ${g}^{(2)}(\tau )$ is proportional to the detection probability of one photon at the time $t$ and a second one at $t+\tau$. ${g}^{(2)}(0)$ is proportional to the detection probability of two photons in the same time. In the case of ${g}^{(2)}(\tau )<{g}^{(2)}(0)$, the detection probability of a second photon after delay time $\tau$ decreases, which corresponds to the bunching effect. When ${g}^{(2)}(\tau )>{g}^{(2)}(0)$, the detection probability of a second photon increases with the delay time, which corresponds to the antibunching effect. For ${g}^{\left(2\right)}\left(\tau \right)=1,\mathrm{ we have the case of coherent states}.$Based on the numerical results, we show the effect of the parameters $q$ and $\chi$ on the statistical properties for the deformed field. Figures 3 and 4 show the comportment of the function ${g}^{(2)}(0)$ versus the time ${\text{T}}$ for the N- and C-configuration, respectively. Generally, the variation of ${g}^{(2)}(0)$ proves that the field statistical properties can provide a different order with respect to the values of $p$ and $\chi$. According to the Figs. 4 and 5, it is visible that ${g}^{(2)}(0)<1$ in the presence of field deformation which mentioned the sub-Poissonian statistics. On the other hand, the existence of Kerr medium leads to enhance classicality of the field indicating the super-Poissonian statistics with ${g}^{\left(2\right)}(0)>1.$

### Quantum entanglement

The entanglement of the FLA–field state can be obtained using the subsystem entropy^57,58^ and it introduced by

16 $$ \begin{gathered} E_{{{\text{field}} - {\text{FLA}}}} = - {\text{Tr}}\left\{ {\rho^{{{\text{FLA}}}} \left( T \right)\ln \left[ {\rho^{{{\text{FLA}}}} \left( T \right)} \right]} \right\}, \hfill \\ = - \mathop \sum \limits_{j = 1}^{4} e_{j} \ln e_{j} ,{ } \hfill \\ \end{gathered} $$

where ${\rho }^{{\text{FLA}}}(T)$ is the atomic density operator given by Eq. (13) and ${e}_{j}$ is the jth eigenvalue of ${\rho }^{{\text{FLA}}}.$

In Figs. 6 and 7, we show the time variation of the atomic entropy of the both configurations considering various values of the parameters $p$ and $\chi$. Generally, the dynamical behavior of the atomic entropy function is widely affected by the parameters $p$ and $\chi$ as well as the atomic configuration. Based on the considered conditions, when we neglect the both effects ($p\to 1$ and $\upchi \to 0$), the function ${S}_{FLA}$ increases from its minimum and then tends to exhibit a structure with rapid oscillations, there is a considerable amount of entanglement, as the time evolves. When the deformation effect is considered ($p\to 2.5$ and $\upchi \to 0$), the FLA–field entanglement is subjected to a change with an increase in the oscillations’ amplitude of the von Neumann entropy. We introduce the Kerr medium effect ($p\to 1$ and $\upchi \to 0.3$), the function ${S}_{{\text{FLA}}}$ increase at the beginning of the interaction with oscillates and then decreases during the dynamics. This indicates that the Kerr medium effect has d destructive effect on the amount the FLA-field entanglement as the time becomes significantly large. On the other hand, the presence of the both effect ($p\to 2.5$ and $\upchi \to 0.3$), the function ${S}_{FLA}$ increases form its minimum value and then randomly as the time evolves. From these results, we note that the deformation effect can enhance the amplitude of entropy oscillations and that the presence of the Kerr medium leads to restrain the entanglement as the time becomes significantly large with a diminution in the oscillations amplitudes. Moreover, the amount of entanglement is more important and the resistance to the Kerr effect is greater in the case of cascade-type.

### Quantum coherence

The off diagonal elementss of the system density operator determine the basic coherence features. The absolute value of the non-diagonal elements is utilized to determine the quantum coherence through the L norm. The $L$-coherence norm is defined as

17 $${C}_{L}=\underset{\varphi \in \mathcal{I}}{{\text{min}}}\parallel \rho -\varphi {\parallel }_{{l}_{1}}=\sum_{l\ne m}\left|{\rho }_{lm}\right|,$$

where $\mathcal{I}$ is the set of incoherent states. The quantum coherence can be quantified using the concept entropy by considering the distance between the state of interest and the closest incoherent state. The relative entropy can be expressed in terms of the von Neumann entropy as

18 $${C}_{R}=S\left(\rho ||{\rho }_{{\text{diag}}}\right)=S\left({\rho }_{{\text{diag}}}\right)-S\left(\rho \right),$$

where ${\rho }_{{\text{diag}}}$ represents the incoherent state. ${C}_{L}$ and ${C}_{R}$ both achieve monotony for all quantum states. In the case of pure state, it is shown that ${C}_{{\text{L}}}$ characterizes the upper bound of ${C}_{R}$.

**Figure 9:**
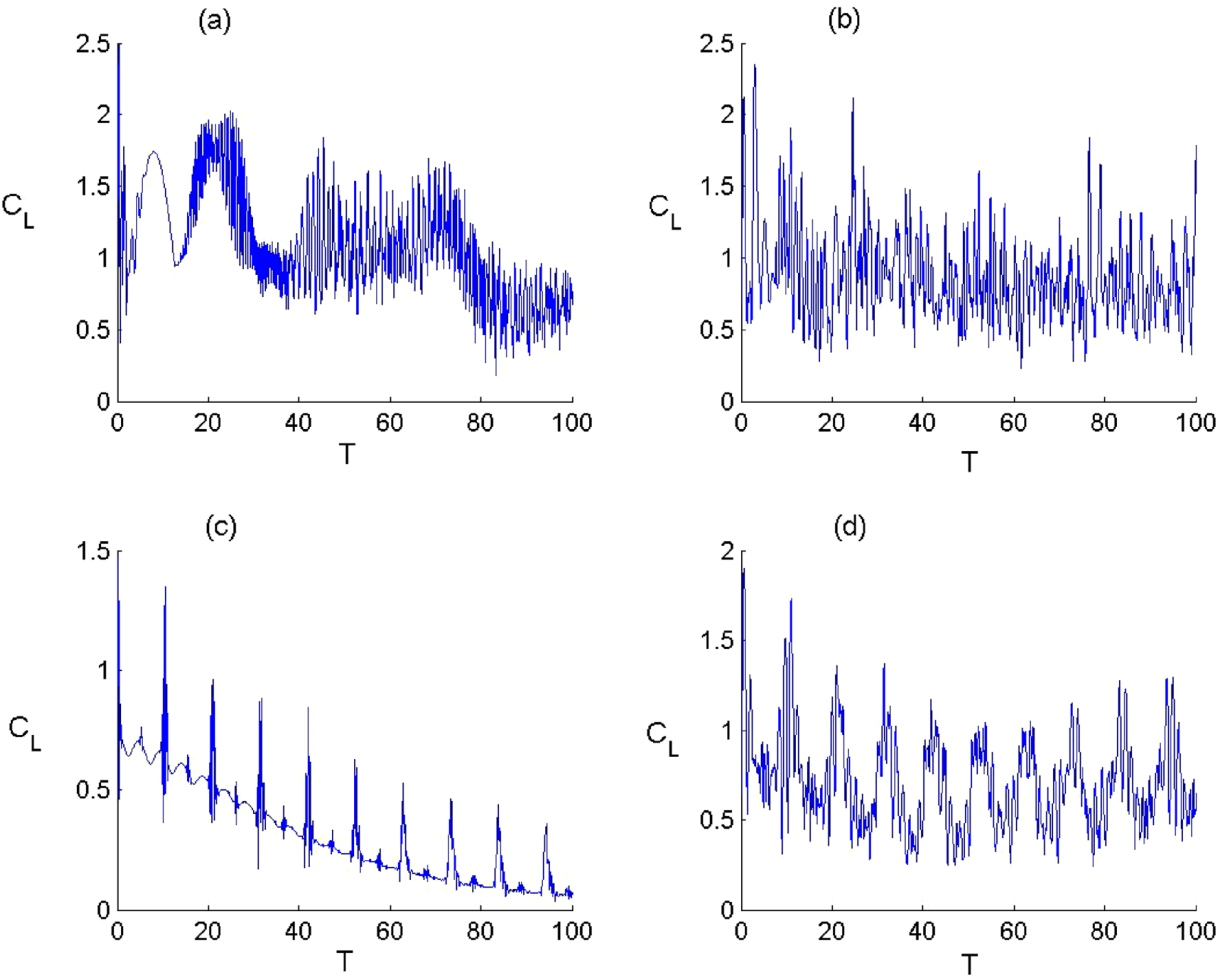
Dynamics of the quantum coherence ${C}_{L}$ of FLA-CC interacting with the field initially in the DCS with $\alpha =\sqrt{20}$ and for the parameter values of (Deformation, Kerr) designed by $\left(p,\chi \right) as$: (**a**) $(p,\chi )=(\mathrm{1,0})$ , (**b**) $(p,\chi )=(\mathrm{2.5,0})$, (**c**) $(p,\chi )=(\mathrm{1,0.3})$ and (**d**) $(p,\chi )=(\mathrm{2.5,0.3})$.

In Figs. 8 and 9, we have displayed the time variation of the atomic coherence according to the values of the parameters $p$ and $\chi$ considering the both configurations. Generally, the dynamical behaviour of the measure of coherence is very sensitive to the nature the quantized field and the Kerr medium, where the coherence measure ${C}_{L}$ oscillates with the time and accompanied by amplitudes and fluctuations that depend on the parameters $p$ and $\chi .$ In the limit of $p\to 1$ and $\upchi \to 0$, the function ${C}_{L}$ tends to exhibit a structure with rapid oscillations. When the deformation effect is considered ($p\to 2.5$ and $\upchi \to 0$), the atomic coherence is subjected to a change with an increase in the oscillations’ amplitude of the function ${C}_{L}$ during the dynamics. When the Kerr medium effect ($p\to 1$ and $\upchi \to 0.3$) is considered, the function ${C}_{L}$ decreases with the time with less oscillations. This indicates that the Kerr medium effect has d destructive effect on the atomic coherence for the both configurations. When the field deformation and Kerr medium are considered ($p\to 2.5$ and $\upchi \to 0.3$), the function ${C}_{L}$ randomly oscillates, where the Kerr medium parameter has a less impact on the amount of coherence during the dynamics. From these results, we conclude that the deformation of field can enhance the amplitude of oscillations in the atomic coherence and that the presence of the Kerr medium leads to organize the behavior of the measure of the quantum coherence accompanied with a diminution in the oscillations amplitudes.

## Conclusion

We have examined quantum entanglement and atomic coherence for a system consisting of a FLA interacted with a nonlinear quantum field. We have assumed that the FLA-field coupling, Kerr medium and quantified field are all $f$-deformed with full nonlinear formalism. We have considered N-configuration and cascade (C)-configuration of the FLA. We have explored the impact of field deformation and Kerr medium on the dynamics of the quantumness measures when the quantized field is initially prepared in a DCS without and with Kerr medium effect. Moreover, we have analyzed the statistical properties of the radiation field using the second order correlation function. The results indicated how the considered quantumness measures in the FLA–field system can be manipulated and controlled through the parameters of the quantum model. In quantum information theory and quantum optics, examining the physical characteristics of the field-atom interaction under ideal circumstances is crucial.

## Data Availability

The datasets used and/or analyzed during the current study are available from the corresponding author on reasonable request.
